# Variability and Stability in Daily Moderate-to-Vigorous Physical Activity among 10 Year Old Children

**DOI:** 10.3390/ijerph120809248

**Published:** 2015-08-07

**Authors:** Sara Pereira, Thayse Natacha Gomes, Alessandra Borges, Daniel Santos, Michele Souza, Fernanda K. dos Santos, Raquel N. Chaves, Peter T. Katzmarzyk, José A. R. Maia

**Affiliations:** 1 CIFI^2^D, Faculty of Sport, University of Porto, Porto 4099-002, Portugal; E-Mails: sara.s.p@hotmail.com (S.P.); thayse_natacha@hotmail.com (T.N.G); borges_alessandra@hotmail.com (A.B.); d.monteiro.santos13@gmail.com (D.S.); mcsouza85@hotmail.com (M.S.); 2 Department of Physical Education and Sports Science, Academic Center of Vitoria, Federal University of Pernambuco, Recife 55608-680, Brazil; E-Mail: fernandak.santos@hotmail.com; 3 Federal University of Technology—Paraná (UTFPR), Campus Curitiba, Curitiba 80230-901, Brazil; E-Mail: raquelnichele@live.com.pt; 4 Pennington Biomedical Research Center, Louisiana State University System, Baton Rouge, LA 70220, USA; E-Mail: peter.katzmarzyk@pbrc.edu

**Keywords:** accelerometry, tracking, patterns, children, Actigraph, recommendations MVPA

## Abstract

Day-to-day variability and stability of children’s physical activity levels across days of the week are not well understood. Our aims were to examine the day-to-day variability of moderate-to-vigorous physical activity (MVPA), to determine factors influencing the day-to-day variability of MVPA and to estimate stability of MVPA in children. The sample comprises 686 Portuguese children (10 years of age). MVPA was assessed with an accelerometer, and BMI was computed from measured height and weight. Daily changes in MVPA and their correlates (gender, BMI, and maturity) were modeled with a multilevel approach, and tracking was calculated using Foulkes & Davies γ. A total of 51.3% of boys and 26.2% of girls achieved 60 min/day of MVPA on average. Daily MVPA was lower during the weekend (23.6% of boys and 13.6% of girls comply with the recommended 60 min/day of MVPA) compared to weekdays (60.8% and 35.4%, boys and girls, respectively). Normal weight children were more active than obese children and no effect was found for biological maturation. Tracking is low in both boys (γ = 0.59 ± 0.01) and girls (γ = 0.56 ± 0.01). Children’s MVPA levels during a week are highly unstable. In summary, boys are more active than girls, maturation does not affect their MVPA, and obese children are less likely to meet 60 min/day of MVPA. These results highlight the importance of providing opportunities for increasing children’s daily MVPA on all days of week, especially on the weekend.

## 1. Introduction

The positive associations between moderate-to-vigorous physical activity (MVPA) and numerous health benefits in children and adolescents has been systematically shown [1,2], namely in bone density increases [3], risk reduction in obesity development [4] as well as in metabolic risk [5,6].

There is some debate about the negative trends in children’s daily physical activity (PA) over the last decades [7,8,9], namely complying with Strong *et al.* [10] and the World Health Organization [1] minimum of 60 min of daily MVPA for individuals aged 5–17 years. For example, Ekelund *et al.* [8] showed no strong evidence to firmly support that objectively measured PA in young people has declined in the last decades, and that between 30% and 40% of youth were sufficiently active.

Recently, Verloigne *et al.* [11], using accelerometry data from five European countries (Belgium, Greece, Hungary, The Netherlands and Switzerland), showed that only 4.6% of girls and 16.8% of boys complied with the 60 min/day of MVPA guideline. In addition, Basterfield *et al.* [12] investigated 2 year changes in PA in English children using accelerometry, and concluded that their overall levels were low. On the contrary, Batista *et al.* [13] found in Portuguese children aged 10–11 years that 51.6% of boys and 22.5% of girls complied with the MVPA guidelines.

It has been shown that demographic, biological, psychological and environmental characteristics are related to variability in PA among children [14,15,16]. Further, gender, body mass index (BMI) [17] and biological maturation [18] are consistently related to mean levels of PA. Recently, a meta-analysis [9] about differences in objectively measured PA in school-aged children suggested higher heterogeneity between studies in MVPA, with 20.3%–53.1% of variance between studies attributable to potential moderating factors.

Physical activity changes over the week, and children tend to be more active during week days than weekend days in some countries [19]. During weekdays, school context characteristics, such as recess time, playground environment, accessibility to game equipment, and physical education classes, affect children’s PA, contributing to the achievement of 60 min of daily MVPA [20,21,22]; whereas during the weekend days children have more leisure time which could be spent in less active pursuits, accumulating less time in MVPA [19]. However, few studies have investigated how children’s PA changes across the entire week, and if there is a pattern in this change. We were able to identify only one study that investigated daily PA levels and patterns during a whole week in children and adolescents 8 to 12 year of age [23]. A pattern for pedometer step counts, and accelerometer-derived MVPA and light PA was characterized, in general, by higher PA on school days (from Monday to Friday), followed by a decrease in PA levels on the weekend. In addition, children were most active on Friday (39% of boys and 21% of girls achieved more than 60 min of MVPA), and least active on Sunday (16% of boys and 10% of girls achieved more than 60 min of MVPA).

Tracking is frequently used to investigate changes in children’s interindividual PA levels, *i.e.*, to describe yearly stability/instability in PA [24]. For example, Dencker *et al.* [25] studied PA changes and stability in 10 years old children over two years, and reported that their PA tracking was low-to-moderate, together with an increase in their time spent in sedentary activities. Although relevant in terms of short-to-long term behaviour stability and changes, these yearly tracking studies do not consider the importance of daily MVPA variation (intraindividual change in interindividual differences) in children’s routines during an important and repeated window of their lives—their weekly routines, governed by the school setting where they spend a large portion of their daily awake time.

Given the paucity of data on day-to-day variability and stability of physical activity, it seems relevant to study children’s compliance with recommended MVPA daily levels, and how it fluctuates across a whole week, as this may prove helpful in identifying more precise intervention windows to better plan and promote more efficient interventions aiming to increase PA levels. Thus, our aims are: (1) to examine the day-to-day variability of MVPA; (2) to determine factors influencing the day-to-day variability of MVPA and (3) to estimate stability of daily MVPA in children.

## 2. Material and Methods

### 2.1. Sample

The sample comprised 686 Portuguese 5th grade children, from elementary school (381 girls and 305 boys), aged 10.5 ± 0.3 years and randomly selected from 23 schools in the Oporto metropolitan area, Portugal. Present data comes from the Portuguese site of the International Study of Childhood Obesity, Lifestyle and the Environment (ISCOLE), a research project conducted in 12 countries from all major world regions. Briefly, this study aims to determine the relationship between lifestyle and obesity in a large multi-national study of children, and to investigate the influence of higher order characteristics such as behavioural settings, physical, social and policy environments on the observed relationships within and between countries [26]. All 5th grade children were invited to take part in ISCOLE, but only those aged between 9 and 11 years old were classified as “eligible” to be part at the project. From those children, a sample of approximately 30–40 children per school was randomly selected (50% for each sex). Non-response was negligible (response rate was 95.7%), and missing information was at random. All children and parents/legal guardians received extensive information regarding the research project; written consent and assent was then obtained from parents/legal guardians and children, respectively. Consents were also obtained from physical education departments, school principals and parental council in each school. The Oporto University Ethics Committee approved the project. Further, all information was collected by certified personnel from the ISCOLE study center under highly controlled conditions as reported elsewhere [26].

### 2.2. Anthropometry

Stature was measured using a Seca 213 portable stadiometer (Hamburg, Germany), without shoes and with the head positioned to the Frankfurt plane, and sitting height was measured while seated on a table with legs hanging freely and arms resting on the thighs.

Body mass was measured with children in light clothing with a portable Tanita SC-240 body composition analyzer (Helligton Heights, IL, USA), which gives reliable and valid information [27]. All procedures were previously described by Katzmarzyk *et al.* [26]. BMI was computed using the standard formula [body mass (kg)/height (m)^2^], and all children were classified as normal weight, overweight or obese according to the cut points defined by the WHO [28]. The cut points adopted by the WHO define overweight as BMI > +1 SD and obese as BMI > +2 SD.

### 2.3. Maturity Offset

Biological maturation was indirectly estimated with the maturity offset procedure proposed by Mirwald *et al.* [29]. This procedure estimates the timing of occurrence of peak height velocity (PHV). The maturity offset estimates the distance each subject is from PHV using chronological age and the value is expressed in decimal years. A positive (+) maturity offset represents the number of years the participant is beyond PHV, whereas a negative (−) maturity offset represents the number of years the subject is before PHV.

### 2.4. Physical Activity

PA was objectively assessed with the Actigraph GT3X+ accelerometer (ActiGraph LLC, Pensacola, FL, USA), with a sampling rate of 80 Hz, during 24 hours/day for nine consecutive days, being only removed during water activities (*i.e.*, showering, swimming).To minimize the effect of reactivity shown in some studies [30,31,32], the first and last day of data assessment were not considered for further analysis, which yielded 7 consecutive days of accelerometer data. Also, there was a random start of monitoring day in the accelerometer use from Monday to Friday.

The accelerometer was attached to the participant using an elastic belt worn around the waist with an adjustable clip. The accelerometer unit was placed in line with the mid-axillary line and lying on the iliac crest (*i.e.*, hip location). ActiLife software was used to download recorded data immediately upon retrieval of each accelerometer. The downloading process produced an AGD file with the following settings: 1 s epoch, 3 axis of orientation, steps, lux (ambient light), inclinometer, and low frequency extension (LFE) [26]. The minimal amount of accelerometer data that was considered acceptable was 4 days with at least 10 h of wear time per day, including at least one weekend day [26]; a total of 686 Children fulfilled this condition. After removal of sleep time, average “awake” wear time was 15.2 h per day. The number of valid cases by day of the week ranged from 647 (94%) on Sunday to 676 on Thursday and Saturday (99%).

Accelerometer data were first divided into awake time and nocturnal sleep time using an automated algorithm [33,34]. After exclusion of the nocturnal sleep period, waking non-wear time was defined as any sequence of at least 20 consecutive minutes of zero activity counts [34]. PA counts were reduced into PA intensity variables using the cut-points of Evenson *et al.* [35]. For the purposes of this study, only time (expressed in minutes) spent in MVPA was used, in each of the seven days (Monday to Sunday). MVPA was defined as greater than 574 activity counts per 15 s.

### 2.5. Data Analysis

Descriptive statistics were computed in SPSS 20. Exploratory analysis showed that MVPA had a highly skewed distribution, and in order to normalize it a square root transformation (Sqrt_MVPA) was used as suggested by Tabachnick and Fidell [36]. Stability (*i.e.*, tracking) of daily MVPA was approached using the γ statistic developed by Foulkes and Davies [37] and implemented in the Timepath software [38]. As an index of tracking, γ is perfect (γ = 1) when a group of individual growth profiles do not intersect, that is when the relative ranking within the response distribution (MVPA) is maintained over time (in our case, over the seven days). On the contrary, no tracking occurs if γ ≤ 0.5; if γ is greater than 0.5, tracking is said to occur. The γ statistic was computed separately for boys and girls. Using a procedure implemented in Timepath software [38,39,40] γ was used in two steps. First, to describe the consistency of MVPA across the seven days, a point estimate of an individual version of γ formulated by Rogosa *et al.* [40] as a measure of individual tracking was used. Since γ was obtained for all participants, the 5th percentile (P5), first quartile (Q1), median (Me), third quartile (Q3), and 95th percentile (P95) of each individual γ were calculated. Secondly, a global γ was estimated to describe children MVPA tracking across the seven days for all subjects.

**Figure 1:**
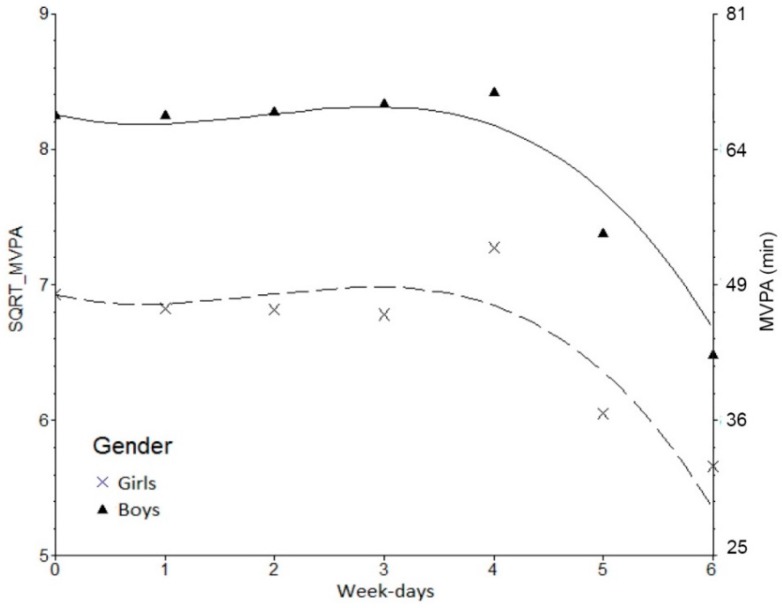
Observed and modelled daily MVPA changes in boys and girls.

Notes: 0 = Monday, 1 = Tuesday, 2 = Wednesday, 3 = Thursday, 4 = Friday, 5 = Saturday, 6 = Sunday.

Daily changes in MVPA and their time-invariant correlates (gender, BMI categories, and maturity offset) were modeled within the SuperMix software v.1 [41] using full maximum likelihood estimation techniques. This was done in a three-step approach. Firstly [Model 1 (M1)], to fit the intricacy of daily changes in MVPA, we used a polynomial function of “time” (*i.e.*, day) with increased complexity. The “time metric” was set as follows: 0 = Monday, 1 = Tuesday, 6 = Sunday as shown below in Figure 1. Guided by SuperMix visual graphic capabilities we fitted a series of nested polynomial models till a 3rd degree. Final decisions about the best fitting solution were made according to deviance and corresponding Chi-square changes in nested models of increasing complexity. A more complex model fits better than a previous one if the differences in their respective deviances are statistically significant. This is done by a Chi-square test with degrees of freedom equal to the difference in estimated parameters of both models. Secondly [Model 2 (M2)], we introduced time-invariant correlates [sex: girls are the reference group, BMI categories (normal weight = reference group, overweight and obese), and maturity offset], and all parameters were simultaneously estimated and tested for their significance. Finally [Model 3 (M3)], we included linear trend (days)-by-sex, and quadratic trend (days^2^)-by-sex interactions. Since a cubic trend (days^3^)-by-sex interaction did not improve the model, we retained the previous one (M3).

## 3. Results

On average, boys and girls have similar (*p* > 0.05) height, weight and BMI; however, as expected, girls are closer (*p* < 0.001) to their age at PHV than boys (Table 1). Further, there is a substantial amount of overweight girls (17.6%) and boys (15.1%), as well as obese (girls = 24.4%; boys = 35.4%).

**Table 1:** Boys and girls basic descriptive statistics (means ± standard deviations, *t* and *p*-values).

	Boys M ± SD	Girls M ± SD	Total M ± SD	*t*	*p*-Value
Height (cm)	143.46 ± 6.42	143.49 ± 7.06	143.47 ± 6.78	0.60	0.952
Weight (kg)	40.52 ± 9.23	40.29 ± 9.23	40.39 ± 9.23	−0.33	0.740
BMI (kg·m^−2^)	19.54 ± 3.36	19.41 ± 3.36	19.47 ± 3.40	−0.51	0.610
Maturity offset (years to PHV)	−2.73 ± 0.43	−1.22 ± 0.53	−1.90 ± 0.89	41.03	<0.001
Weight status	n (%)	n (%)	n (%)	χ^2^	*p*-value
Normal weight	151 (49.5%)	221 (58.0%)	372 (54.2%)	2.899	0.004
Overweight	46 (15.1%)	67 (17.6%)	113 (16.5%)		
Obese	108 (35.4%)	93 (24.4%)	201 (29.3%)		

Table 2 shows medians (Mdn), Interquartile Range (P25 and P75) and means (M ± SD) of time spent in MVPA, as well as the frequency of boys and girls complying with daily recommended 60 min of MVPA for each of the days. On average, boys have higher MVPA than girls. Further, during the weekdays, time spent in MVPA ranges from 37.5 to 69.5 min and 28.75 to 53 min in boys and girls, respectively. During the weekend, a pronounced decrease in MVPA occurs. The percentage of boys and girls meeting daily MVPA 60 min recommendations is moderate. Values are higher during the week and lower on the weekend (as can be seen on Table 2, where the number of the valid cases, e.g., children, are presented for each day of the week). 

**Table 2:** Descriptive statistics for boys and girls daily MVPA.

	Monday	Tuesday	Wednesday	Thursday	Friday	Saturday	Sunday
**Boys**							
Mdn (IQR)	68.88 (49.38–91.88)	67.00 (48.25–92.25)	68.50 (48.50–91.00)	69.50 (47.50–97.50)	68.75 (49.75–94.50)	50.00 (32.25–82.50)	37.50 (22.50–63.50)
M (SD)	71.88 (32.60)	72.45 (35.60)	72.63 (33.83)	73.90 (35.26)	74.52 (33.78)	60.64 (38.87)	47.36 (33.53)
CV	0.45	0.49	0.47	0.48	0.45	0.64	0.71
Girls							
Mdn (IQR)	48.88 (32.75–63.25)	46.25 (31.75–63.25)	46.75 (33.50–62.00)	44.25 (30.50–64.25)	53.00 (38.25–67.50)	35.00 (22.25–51.25)	28.75 (18.00–45.00)
M (SD)	50.84 (24.17)	49.52 (24.42)	48.95 (22.24)	48.87 (23.94)	55.67 (25.77)	40.04 (24.20)	35.69 (25.44)
CV	0.48	0.49	0.45	0.49	0.46	0.60	0.71
%Rb	60.8%	59.5%	60.5%	59.2%	60.5%	42.1%	28.4%
%Rg	28.4%	28.8%	27.7%	28.4%	36.6%	18.3%	14.9%

Mdn (IQR): Median (min/day) and interquartile range; M (SD): Means (min/day) and standard deviations; CV: coefficient of variation; %Rb: Percentage of boys reaching daily recommended 60 min of MVPA; %Rg: Percentage of girls reaching daily recommended 60 min of MVPA.

Daily modeling results of Sqrt_MVPA are shown in Table 3, which presents the best fitting polynomial model (M1: 3rd degree), M2 with gender, BMI categories, and maturity off-set as covariates, and M3 with the two interactions [linear trend (days)-by-sex and quadratic trend (days^2^)-by-sex]. M1 with a 3rd degree polynomial shows a nonlinear tendency in MVPA, with a clear declining trend starting on Friday and reaching the lowest values on Sunday. As shown in M2, boys have, on average, more daily minutes of Sqrt_MVPA than girls (β = 1.64 ± 0.21, *p* < 0.001) and obese children spent less daily Sqrt_MVPA minutes than normal weight (β = −0.42 ± 0.14, *p* = 0.002). Figure 1 shows clearly this difference between boys and girls. No significant effects were noticed for biological maturation on Sqrt_MVPA. In M3, a significant effect is visible in the quadratic trend (days^2^)-by-sex interaction ((β = −0.04 ± 0.01, *p* = 0.005).

**Table 3:** Parameter estimates (± standard-errors) and *p*-values of the three models.

	Model 1	Model 2	Model 3
***Fixed effects***			
Intercept	7.515 ± 0075, *p* < 0.001	7.204 ± 0.189, *p* < 0.001	7.207 ± 0.194, *p* < 0.001
Linear	−0.194 ± 0.093, *p* = 0.037	−0.195 ± 0.092, *p* = 0.036	−0.263 ± 0.010, *p* = 0.008
Quadratic	0.153 ± 0.037, *p* < 0.001	0.153 ± 0.037, *p* < 0.001	0.170 ± 0.038, *p* < 0.001
Cubic	−0.027 ± 0.004, *p* < 0.001	−0.027 ± 0.004, *p* < 0.001	−0.027 ± 0.004, *p* < 0.001
Sex		1.588 ± 0.204, *p* < 0.001	1.580 ± 0.224, *p* < 0.001
Overweight		−0.077 ± 0.138, *p* = 0.574	−0.079 ± 0.138, *p* = 0.567
Obese		−0.418 ± 0.130, *p* = 0.001	−0.417 ± 0.130, *p* = 0.001
Maturity offset		0.136 ± 0.116, *p* = 0.239	0.136 ± 0.116, *p* = 0.240
Linear-by-Sex			0.155 ± 0.084, *p* = 0.067
Quadratic-by-Sex			−0.038 ± 0.013, *p* = 0.004
***Variance components***			
Intercept	1.492 ± 0.111, *p* < 0.001	1.017 ± 0.863, *p* < 0.001	1.14 ± 0.10, *p* < 0.001
Linear	0.015 ± 0.004, *p* = 0.051	0.019 ± 0.044, *p* < 0.001	0.02 ± 0.01, *p* = 0.110
Residual	2.462 ± 0.058	2.450 ± 0.058	2.39 ± 0.06
Deviance	18,690.16	18,506.24	18,489.41
N° of estimated parameters	7	11	13

Foulkes and Davies γ distributional values in boys’ and girls’ weekly Sqrt_MVPA showed that γ ≥ 0.5 provides evidence of tracking, about 50% of boys and girls do show stability in their daily Sqrt_MVPA levels. Boys seem to be more stable, *i.e.*, their γ values are always higher. Population γ estimates (interindividual differences in MVPA consistency across the all week) showed low to no tracking (girls: γ = 0.56 ± 0.01; boys γ = 0.59 ± 0.01).

## 4. Discussion

The present study aimed: (1) to examine the day-to-day variability of MVPA, (2) to determine factors influencing the day-to-day variability of MVPA and (3) to estimate stability in daily MVPA in children.

In the present study ≈60% of the boys and ≈30% of the girls met the daily MVPA guidelines during the week, whereas during the weekend these values decreased to 42.1% and 18.3% on Saturday, and 28.4% and 14.9% on Sunday, respectively. Similar results were found in a previous report with 10–11 years old Portuguese children, where using a different cut-off to define MVPA, the authors indicated that during the seven days, 51.6% of the boys and 22.5% of the girls met the WHO recommendations [1]. Regarding differences between week days and weekend days, previous studies in Portuguese children showed different results. For example, Vale *et al.*, studying children aged 2–6 years reported that 93.5% of them met the daily MVPA during the week days, and this percentage declined to 77.6% during weekend [42]. On the other hand, Laguna *et al.* [43], studying MVPA in 9-y old Portuguese children, found different results, where children spent more time in MVPA during the weekend when compared to week days. Further, in a sample of primary school children aged 5–10 years from southwest Germany, Kettner *et al.* [44] found that 68 % of the boys and 28 % of the girls met the recommendations of 60 min/day of MVPA and higher PA levels were observed during the week, which is in accordance with our finds. This difference in the results among studies can be linked not only to sample characteristics but also to the use of different cut-points to define MVPA, as well as different accelerometer models and data reduction procedures, as previously suggested [45]. 

Differences between weekday and weekend MVPA may be attributed to the longer time children spend in sedentary behaviours (e.g., more screen time) on weekends [46,47,48]. It is also possible that these differences may be associated with peer and family support for PA [49,50], since there is some evidence that families play an important role in children’s PA and sedentary behaviour [51,52]. For example, Jago, *et al.* [53] reported that parental TV-viewing time was associated with children’s TV-viewing time in a study of 7–10 years old Portuguese children. Moreover, Vander *et al.* [54] found that parental beliefs and support are correlated with prevention strategies to increase children’s PA among 10 and 11 years old Canadian children, especially during the weekend. These studies reinforce, in different ways, the role of parents on children’s PA. In Australian children followed longitudinally from 8 to 12 years, using the same method to estimate PA, as well as the same cut-points, used in our study, similar trends were found, with a decline in PA on the weekends [23]. However, the percentage of Australian children achieving WHO guidelines was lower than in Portuguese children, ranging from 30% to 40% in boys on weekdays, and below 22% on weekends, while in girls this percentage ranges from 15% to 21% on weekdays, and 11% on weekends. 

These differences between Portuguese and Australian children may be due to different sports policies implemented in the school settings. For example, within the Portuguese school system all children have at least two days of physical education per week, which represents 135 min of structured activity, and have the opportunity to freely practice sports in club settings outside the classroom with a varied range of forms available (soccer, basketball, badminton, tennis, swimming, gymnastics, *etc.*). In Australia, 120 min per week of physical education is mandated in most states, however, not all schools actually schedule 120 min [55,56]. Out of school hours sport is sometimes offered by schools, but more commonly by non-school-based clubs. Since we did not have any additional information, and because the samples from these two studies have cultural and social differences, we tried to speculate about possible reasons for this discrepancy in the results. 

Our multilevel modelling results indicated a nonlinear trend in MVPA levels with a very modest increase during schooldays and a rapid decline from Friday to Sunday. Moreover, both gender and BMI category (between obese children and normal weight) were found to have significant effects on MVPA levels. Results for daily PA (Table 2) show that although boys and girls have similar weekly MVPA patterns, boys spend more time in MVPA than girls, regardless of the day, which is in accordance with sex-differences observed in PA among youth [57]. It has been suggested that boys tend to be more active than girls due to biological factors [58], but this may also be attributable to parental, social and environmental factors [57], which imply more time engaging in sports and free-living activities. Furthermore, boys typically view school breaks as a chance to engage in competitive games that tend to dominate play spaces in the school yard while girls view the school break period as a time for socializing [17], *i.e.*, the majority of boys manifest competency by being active players in sports games and the majority of girls walk and talk [59]. On the other hand, throughout the after-school period there is some suggestion that parents perceive the neighborhood to be safer for adolescent boys compared to adolescent girls [60], given boys one more possibility to be physically active. 

Obese children in this study were less active than their normal weight peers, but no MVPA differences between overweight and normal weight children were found, which is consistent with previous data in preschool-aged children [61] although it is possible that the adiposity rebound in preschool children may condition their MVPA [62,63]. It is possible that higher self-efficacy of normal weight/overweight children as compared to obese children may explain this issue. It has been reported that obese children have a diminished notion of their body capabilities which are linked to lower motor coordination and skill development and consequent MVPA levels [64,65,66]. Thus, this group of children should be followed with particular care since they tend to be less active during adolescence and have a higher probability of developing cardiometabolic diseases [67]. Biological maturation had no significant effect on MVPA levels across the week in this study, which agrees with data reported by Wickel *et al.* [68] comparing PA levels among early, average, and late maturing boys and girls, and after controlling for chronological age. However, Thompson *et al.* [69], using data from a seven year longitudinal study (participants were 9–18 years of age), showed that PA decreased with increasing biological age, with more mature children being less physically active. In addition, since girls mature earlier than boys, this might explain some of the observed difference between genders. Although in our data there was a significant mean difference in maturity offset favoring girls (see Table 1), maturity was not a significant PA predictor which may be related to the narrow age range of the sample (9 to 11 years), in which only some girls are prepubescent.

One of the novelties of the present study is the use of tracking to understand how stable children’s daily MVPA patterns are over a week. Similar approaches have been used to understand interindividual differences in intraindividual PA changes, but only between consecutive years or over several years [25,70]. These studies indicate that children’s and adolescent’s PA levels have low to moderate tracking [25,70]. However, we were not able to identify any study that investigated heterogeneity in MVPA levels over a week in children, as this time frame is generally used with accelerometry data. The main finding in our data is that only 5% of children showed high tracking (Boys γ = 0.85; Girls γ = 0.87), *i.e.*, were systematically stable in their MVPA trajectories over a week. This is somewhat surprising and should not be confused with results from Table 2 and Table 3. In fact, a child may reach the cutpoint of 60 min a day of MVPA, but his/her results across the week may fluctuate in some random fashion, governed by school activities and/or leisure activities outside the school. It is also possible that changes in children’s routines, from week to weekend, may contribute to this low tracking observed; at this context, during the week days children spend most of their time at school, getting more opportunities to be engaged in structured PA but with less time to be engaged in free PA; on the contrary, during the weekends, children have more free time that can be used to get engaged in free PA, but not in structured ones. 

Further analysis using latent trajectories or latent classes should be used to identify different groups of children and potential covariates that may condition their belonging to low and high tracking groups in order to improve the efficacy of intervention programs.

This study has some limitations. Firstly, the cross-sectional design does not allow cause-and-effect interpretation. Secondly, biological maturation was indirectly estimated with the maturity offset method and this procedure has never been validated in Portuguese Children. Nevertheless, other common biological markers of maturity also have similar problems, namely financial (in the case with x-ray methods) as well as ethical (for instance in sexual maturation characteristics) and have never been systematically cross-validated to other countries or cultures. Thirdly, although our time frame is only seven days, and our approach is mostly based on MVPA trends across the week *i.e.*, day-to-day variability and stability, as well as on interindividual differences, it would be of interest in the future to study more deeply the possible determinants of those who are more consistent (high trackers) and less so (low trackers) in their daily MVPA. Notwithstanding these limitations, the study has several important strengths. Firstly, the use of an objective method to estimate MVPA. Secondly, the large sample size that provides detailed information about a particular age group. Thirdly, the use of sophisticated statistical procedures to analyze day-to-day variability and stability on MVPA over the week and the use of standardized methods of data collection within a robust quality control program [26].

## 5. Conclusions 

In conclusion, the results of this study provide further support for the known influence of gender and weight categories on children’s MVPA. Daily MVPA has a nonlinear trend with a marked decrease during the weekend. Explicitly, girls and obese children spend fewer minutes per day in MVPA. Further, maturation does not affect 10 years old children’s MPVA although girls are, on average, at the take-off of their growth spurt. A strong instability in MVPA levels was observed over a week. This set of results raises new questions about the importance of school settings and household effects on children’s MVPA.
